# A Manual of Medical Jurisprudence

**Published:** 1888-02

**Authors:** 


### BOOK NOTICES.

A MANUAL OF MEDICAL JURISPRUDENCE, with special reference to Diseases and Injuries of the Nervous System. By Allan McLane Hamilton, M. D. ; pp. 390. Price, $2.75. New York: E. B. Treat, 1887.

As, unfortunately, all physicians are liable, at any time, to be dragged into legal squabbles, of one kind or another, this treatise of Dr. Hamilton will be of service to us all. It is simply a concise and well-arranged reference book for lawyers and doctors, with reference to those conditions of the nervous system which are apt to be under discussion in our courts. A large amount of useful hints will be found in this little volume.

